# A Finite-Time Sliding-Mode Controller Based on the Disturbance Observer and Neural Network for Hysteretic Systems with Application in Piezoelectric Actuators

**DOI:** 10.3390/s23146246

**Published:** 2023-07-08

**Authors:** Liqun Cheng, Wanzhong Chen, Liguo Tian, Ying Xie

**Affiliations:** 1 College of Communication Engineering, Jilin University, Changchun 130012, China; chengliqun@cust.edu.cn; 2 International Research Centre for Nano Handling and Manufacturing of China, Changchun University of Science and Technology, Changchun 130022, China; tlg@cust.edu.cn (L.T.); xiey@cust.edu.cn (Y.X.)

**Keywords:** piezoelectric actuator, finite-time stable, radial basis function, sliding-mode controller, disturbance observer

## Abstract

Piezoelectric actuators (PEAs) have the benefits of a high-resolution and high-frequency response and are widely applied in the field of micro-/nano-high-precision positioning. However, PEAs undergo nonlinear hysteresis between input voltage and output displacement, owing to the properties of materials. In addition, the input frequency can also influence the hysteresis response of PEAs. Research on tracking the control of PEAs by using various adaptive controllers has been a hot topic. This paper presents a finite-time sliding-mode controller (SMC) based on the disturbance observer (DOB) and a radial basis function (RBF) neural network (NN) (RBF-NN). RBF-NN is used to replace the hysteresis model of the dynamic system, and a novel finite-time adaptive DOB is proposed to estimate the disturbances of the system. By using RBF-NN, it is no longer necessary to establish the hysteresis model. The proposed DOB does not rely on any priori knowledge of disturbances and has a simple structure. All the solutions of closed-loop systems are practical finite-time-stable, and tracking errors can converge to a small neighborhood of zero in a finite time. The proposed control method was compiled in C language in the VC++ environment. A series of comparative experiments were conducted on a platform of a commercial PEA to validate the method. According to the experimental results of the sinusoidal and triangular trajectories under the frequencies of 1, 50, 100, and 200 Hz, the proposed control method is feasible and effective in improving the tracking control accuracy of the PEA platform.

## 1. Introduction

Piezoelectric actuators (PEAs) have the properties of high bandwidth, powerful output, and fast response. They have been widely used in precision manufacturing [1], micro-electromechanical systems (MEMS) [2], micro-/nano-instruments [3,4], atomic force microscopy (AFM) [5,6], scanning tunneling microscopy (STM) [7,8], adaptive optics [9], etc. PEAs are generally manufactured from barium titanate or lead zirconate titanate (PZT). Because of the natural properties of these materials, PEAs undergo nonlinear hysteresis between input voltage and output displacement [10,11,12]. In addition, the input frequency can also influence the hysteresis response of PEAs (i.e., their rate-dependent properties). Classic hysteresis models are Krasnosel’skii–Pokrovkii (KP) [10], Prandtl–Ishlinskii (PI) [10,13,14], Preisach [4,15], Backlash-like [10] and Bouc–Wen (BW) [16,17]. Hysteresis models can be used as feedforward controllers or combined feedforward–feedback controllers [10,17]. In recent years, research on reducing the errors of PEAs by using various control methods has been a hot topic [10,17,18,19].

A feedforward–feedback controller was proposed in [20]. This hybrid controller is a combination of the PI model and $H_{\infty }$ controller. The PI model and $H_{\infty }$ controller are used as feedforward and feedback controllers, respectively. Janaideh et al. [21] proposed a hybrid controller of the PI model and internal model-based feedback control to compensate for the piezoelectric cantilever actuator. Al-Nadawi et al. [22] proposed an inversion-free compensator via adaptive conditional servomechanism, in which the compensator was a PI-model-based adaptive servo controller. In [23], a hybrid feedforward–feedback compensator based on a nonlinear auto-regressive exogenous (NARX) model and proportional–integral–differential (PID) control was proposed. Wang et al. [24] proposed an adaptive nonsingular terminal sliding-mode controller (ATSMC) for a PEA system. In [24], the nonsingular mode was chosen as the sliding surface, and the adaptive gain was added. An adaptive observer-based integral SMC was proposed in [25], for which the adaptive observer was combined with a hysteresis-state observer and an adaptive-gain observer.

Many studies have been carried out on using neural networks (NNs) to construct controllers. An inversion-free predictive controller based on a neural network (NN) nonlinear model was proposed in [26]. Liu et al. [27] proposed a predictive controller based on the NN model. Takagi–Sugeno fuzzy NN hysteresis modeling for shape memory alloy was proposed in [28]. Xu and Zhou used the Elman NN to calculate the parameters of the KP model [29]. Lin et al. [30] proposed a sliding-mode controller (SMC)-based error estimator of recurrent NNs. They used an NN to estimate the uncertainty. In [31], an adaptive NN was used to estimate the time delay function of the nonlinear system-based surface control.

Disturbance observers (DOBs) as tools to improve the accuracy of nonlinear systems have been widely used in robot manipulators [32], ships [33], spacecraft [34], quadrotors [35], etc. Recently, various DOBs have been developed in enhancing the control accuracy of PEAs. In [36], a combination of uncertainty and disturbance estimators and a PI model was proposed to achieve high-precision output tracking of a piezoelectric actuator. In [37], a BW model-based SMC that included a disturbance estimation part was proposed. A sliding-mode DOB-based SMC control mechanism for surgical devices was proposed in [38]. Zhang et al. [39] proposed a DOB-based adaptive controller to control rate-dependent hysteresis systems. A polynomial model-based fractional-order DOB was proposed in [40]. The proposed method can compensate for and track the high-frequency displacements of PEAs.

Inspired by the above works, a finite-time SMC based on the finite-time DOB and radial basis function (RBF) NN (RBF-NN) is proposed in this paper. To the best of our knowledge, this is the first work on a novel finite-time DOB and RBF-NN control method of nonlinear systems with both hysteresis and rate dependence. The main contributions of the paper are summarized as follows: First, an RBF-NN is used to replace the nonlinear hysteresis model of the PEA dynamic system, which has the advantages of a simple structure, and the inversion of the nonlinear model does not need to be calculated. Second, a novel finite-time adaptive DOB is proposed to estimate the disturbances of the system. Compared with other DOBs, the proposed DOB does not rely on any priori knowledge of disturbances and has a simple structure. Third, the updated rules of the RBF-NN and proposed DOB guarantee the errors of DOB and ensure that the RBF-NN is stable and bounded. Fourth, based on the finite-time stability method, the proposed inversion-free SMC-based finite-time DOB and RBF-NN can ensure that the system errors converge to a small neighborhood of zero in a finite time. A series of comparative experiments show that the proposed finite-time SMC has high accuracy and good robustness in the tracking control of the trajectories under both high and low frequencies.

The rest of the paper is organized as follows: Section 2 introduces the preliminaries. In Section 3, the updated rules of the RBF-NN and proposed DOB are constructed and the proposed finite-time SMC is designed. Section 4 presents the experimental setup and results. Conclusions are given in Section 5.

## 2. Preliminaries

This section presents some preparations before the finite-time observer and SMC design, including some lemmas, the formula of RBF-NN, and the model of the system.

**Lemma** **1** ([33])**.** *For any real numbers* $g_{i}$*,* i=1,…,n *and* 0<c<1*, the following inequality holds:*

(1) $${\left(\left|g_{1}\right|+\ldots +\left|g_{n}\right|\right)}^{c}\leq {\left|g_{1}\right|}^{c}+\ldots +{\left|g_{n}\right|}^{c}$$

**Lemma** **2** ([34])**.** *If the Lyapunov function* V(x(t))  *satisfies the following inequality*

(2) $$\dot{V}(x(t))\leq -pV^{\frac{1}{2}}(x(t))+q$$

*where* p*,* q>0 *are two constants, then the system is finite-time-stable, and the finite-time satisfies the following equation* $T\leq \frac{2V^{\frac{1}{2}}(t_{0})}{\theta_{0}p}$*,* $t_{0}$ *is the initial time,* $0<\theta_{0}<1$.

The RBF-NN is formulated as

(3) $$n(X)=W^{T}h(X^{*})$$

where $W={\left[\begin{array}{cccc} W_{1} & W_{2} & \ldots & W_{m} \end{array}\right]}^{T}\in R^{m}$ is the ideal weight, m is the number of the hidden layer of NN, $h(X^{*})={\left[\begin{array}{cccc} h_{1}(X^{*}) & h_{2}(X^{*}) & \ldots & h_{m}(X^{*}) \end{array}\right]}^{T}\in R^{m}$, and $X^{*}$ is the input vector. $h_{i}(X^{*})$ can be chosen as a Gaussian function, which is in the following equation, $h_{i}(X^{*})=\exp\left[\frac{-{\left(X^{*}-\mu \right)}^{T}(X^{*}-\mu )}{2\varepsilon^{2}}\right]$, i=1,2,…,n, where $\mu ={\left[\begin{array}{cccc} \mu_{1} & \mu_{2} & \ldots & \mu_{n} \end{array}\right]}^{T}$ is the central vector, ε is the width of the Gaussian function, and the range of $h_{i}(X^{*})$ is $0<h(X^{*})\leq 1$.

The dynamic model of the system is defined as follows [24]:

(4) $$m\dot{x}+bx+f=ku+d$$

where m is the mass of PEA, and b is the damping coefficient. The term k is the electromechanical ratio, and u is the control input of the system. $\dot{x}$ and x are the velocity and position, respectively. The function f represents the hysteresis model, and d is the disturbance of the system.

## 3. Adaptive Finite-Time DOB and SMC Design

The dynamic model (4) is rewritten using Equation (5):

(5) $$\dot{x}=k_{m}u-b_{m}x-f_{m}+d_{m}$$

where $k_{m}=\frac{k}{m}$, $b_{m}=\frac{b}{m}$, $f_{m}=\frac{f}{m}$, $d_{m}=\frac{d}{m}$.

The tracking error is defined as follows:

(6) $$e=x-x_{d}$$

where $x_{d}$ is the desired position. Then, the derivative of error is defined as follows:

(7) $$\dot{e}=k_{m}u-b_{m}x-f_{m}+d_{m}-{\dot{x}}_{d}$$

To design adaptive finite-time DOB, the variable σ is introduced first as follows:

(8) σ=ω−e

where ω is designed as

(9) $$\dot{\omega }=k_{m}u-b_{m}x-{\hat{f}}_{m}+{\hat{d}}_{m}-{\dot{x}}_{d}$$

${\hat{f}}_{m}$ and ${\hat{d}}_{m}$ are the estimations of the hysteresis model $f_{m}$ and disturbance $d_{m}$ respectively.

The estimated hysteresis model ${\hat{f}}_{m}$ is replaced by RBF-NN, which is given by

(10) $${\hat{f}}_{m}={\hat{W}}^{T}h$$

where h is the hidden layer. The hysteresis model $f_{m}$ in Equation (7) is replaced by RBF-NN, and defined as $f_{m}=W^{T}h$. $\hat{W}$ is the estimation of weight W, and the update of $\hat{W}$ is defined as

(11) $$\dot{\hat{W}}=(-\delta_{2}\hat{W}+\sigma h)$$

where $\delta_{2}>0$.

**Remark** **1.** *All the terms in Equation (5) are bounded. Then, the* $d_{m}$ *is bounded and denoted as* $\left|d_{m}\right|\leq \tau$.

The estimation $d_{m}$ is given by

(12) $${\hat{d}}_{m}=-k_{1}\sigma -\hat{\tau }\frac{\sigma }{\left|\sigma \right|}-k_{2}\frac{\sigma }{\left|\sigma \right|}$$

where $k_{1}$ and $k_{2}$ are positive gains, and $\hat{\tau }$ is the estimation of τ. The update $\hat{\tau }$ is designed as

(13) $$\dot{\hat{\tau }}=(-\delta_{1}\hat{\tau }+\left|\sigma \right|)$$

where $\delta_{1}>0$. The estimated errors of the weights of RBF, disturbance, and τ are defined as follows:

(14) $$\tilde{W}=W-\hat{W}$$

(15) $${\tilde{d}}_{m}=d_{m}-{\hat{d}}_{m}$$

(16) $$\tilde{\tau }=\tau -\hat{\tau }$$

**Lemma** **3.** Considering the nonlinear system (7), the adaptive finite-time DOB is designed as Equations (8)–(13). Then, ${\hat{d}}_{m}$ will converge to the neighborhood of $d_{m}$ in a finite time.

**Proof.**  Consider the Lyapunov function candidate for the system as follows:

(17) $$V_{1}=\frac{1}{2}\sigma^{2}+\frac{1}{2}{\tilde{\tau }}^{2}+\frac{1}{2}{\tilde{W}}^{T}\tilde{W}$$

The time derivative $V_{1}$ is given by

(18) $$\begin{array}{ll} {\dot{V}}_{1} & =\sigma \dot{\sigma }-\tilde{\tau }\dot{\hat{\tau }}-{\tilde{W}}^{T}\dot{\hat{W}}\\ & =\sigma (\dot{\omega }-\dot{e})-\tilde{\tau }\dot{\hat{\tau }}-{\tilde{W}}^{T}\dot{\hat{W}}\\ & =\sigma (f_{m}-{\hat{f}}_{m}+{\hat{d}}_{m}-d_{m})-\tilde{\tau }\dot{\hat{\tau }}-{\tilde{W}}^{T}\dot{\hat{W}}\\ & =\sigma (W^{T}h-{\hat{W}}^{T}h+{\hat{d}}_{m}-d_{m})-\tilde{\tau }\dot{\hat{\tau }}-{\tilde{W}}^{T}\dot{\hat{W}}\\ & =\sigma ({\tilde{W}}^{T}h-k_{1}\sigma -\hat{\tau }\frac{\sigma }{\left|\sigma \right|}-k_{2}\frac{\sigma }{\left|\sigma \right|}-d_{m})-\tilde{\tau }\dot{\hat{\tau }}-{\tilde{W}}^{T}\dot{\hat{W}}\\ & \leq \sigma {\tilde{W}}^{T}h-k_{1}\sigma^{2}-\hat{\tau }\left|\sigma \right|-k_{2}\left|\sigma \right|+\left|\sigma \right|\left|d_{m}\right|-\tilde{\tau }(-\delta_{1}\hat{\tau }+\left|\sigma \right|)-{\tilde{W}}^{T}(-\delta_{2}\hat{W}+\sigma h)\\ & \leq -k_{1}\sigma^{2}-k_{2}\left|\sigma \right|-\hat{\tau }\left|\sigma \right|+\left|\sigma \right|\tau -\tilde{\tau }\left|\sigma \right|+\delta_{1}\tilde{\tau }\hat{\tau }+\delta_{2}{\tilde{W}}^{T}\hat{W}\\ & \leq -k_{1}\sigma^{2}-k_{2}\left|\sigma \right|+\tilde{\tau }\left|\sigma \right|-\tilde{\tau }\left|\sigma \right|+\delta_{1}\tilde{\tau }\hat{\tau }+\delta_{2}{\tilde{W}}^{T}\hat{W}\\ & \leq -k_{1}\sigma^{2}-k_{2}\left|\sigma \right|+\delta_{1}\tilde{\tau }\hat{\tau }+\delta_{2}{\tilde{W}}^{T}\hat{W}\\ & \leq -k_{2}\left|\sigma \right|+\delta_{1}\tilde{\tau }\hat{\tau }+\delta_{2}{\tilde{W}}^{T}\hat{W} \end{array}$$

Note that

(19) $$\begin{array}{ll} \tilde{\tau }\hat{\tau } & =\tilde{\tau }(\tau -\tilde{\tau })\\ & \leq \frac{1}{2}{\tilde{\tau }}^{2}+\frac{1}{2}\tau^{2}-{\tilde{\tau }}^{2}\\ & =-\frac{1}{2}{\tilde{\tau }}^{2}+\frac{1}{2}\tau^{2} \end{array}$$

(20) $$\begin{array}{ll} {\tilde{W}}^{T}\hat{W} & ={\tilde{W}}^{T}(W-\tilde{W})\\ & \leq \frac{1}{2}{\tilde{W}}^{T}\tilde{W}+\frac{1}{2}W^{T}W-{\tilde{W}}^{T}\tilde{W}\\ & =-\frac{1}{2}{\tilde{W}}^{T}\tilde{W}+\frac{1}{2}W^{T}W \end{array}$$

Substituting (19) and (20) into (18) yields

(21) $$\begin{array}{ll} V_{1} & \leq -k_{2}\left|\sigma \right|-\frac{\delta_{1}}{2}{\tilde{\tau }}^{2}+\frac{\delta_{1}}{2}\tau^{2}-\frac{\delta_{2}}{2}{\tilde{W}}^{T}\tilde{W}+\frac{\delta_{2}}{2}W^{T}W\\ & =-k_{2}\left|\sigma \right|-\frac{\delta_{1}}{2}{\left({\tilde{\tau }}^{2}\right)}^{\frac{1}{2}}-\frac{\delta_{2}}{2}{\left({\tilde{W}}^{T}\tilde{W}\right)}^{\frac{1}{2}}-\frac{\delta_{1}}{2}{\tilde{\tau }}^{2}+\frac{\delta_{1}}{2}\tau^{2}+\frac{\delta_{1}}{2}{\left({\tilde{\tau }}^{2}\right)}^{\frac{1}{2}}-\frac{\delta_{2}}{2}{\tilde{W}}^{T}\tilde{W}+\frac{\delta_{2}}{2}W^{T}W+\frac{\delta_{2}}{2}{\left({\tilde{W}}^{T}\tilde{W}\right)}^{\frac{1}{2}} \end{array}$$

The terms $-\frac{\delta_{1}}{2}{\tilde{\tau }}^{2}+\frac{\delta_{1}}{2}\tau^{2}+\frac{\delta_{1}}{2}{\left({\tilde{\tau }}^{2}\right)}^{\frac{1}{2}}$ and $-\frac{\delta_{2}}{2}{\tilde{W}}^{T}\tilde{W}+\frac{\delta_{2}}{2}W^{T}W+\frac{\delta_{2}}{2}{\left({\tilde{W}}^{T}\tilde{W}\right)}^{\frac{1}{2}}$ in (21) are quadratic functions and have the upper bounds $\lambda_{1}$ and $\lambda_{2}$, respectively [32]. The upper bounds are defined as

(22) $$\lambda_{1}=\frac{\delta_{1}(1+4\tau^{2})}{8}$$

(23) $$\lambda_{2}=\frac{\delta_{2}(1+4W^{T}W)}{8}$$

Using Lemma 1 and upper bounds, Equation (21) can be rewritten as follows:

(24) $$\begin{array}{ll} V_{1} & \leq -k_{2}\left|\sigma \right|-\frac{\delta_{1}}{2}{\left({\tilde{\tau }}^{2}\right)}^{\frac{1}{2}}-\frac{\delta_{2}}{2}{\left({\tilde{W}}^{T}\tilde{W}\right)}^{\frac{1}{2}}+\lambda_{1}+\lambda_{2}\\ & \leq -\min(\sqrt{2}k_{2},\frac{\sqrt{2}}{2}\delta_{1},\frac{\sqrt{2}}{2}\delta_{2})(\frac{1}{\sqrt{2}}\left|\sigma \right|+\frac{1}{\sqrt{2}}{\left({\tilde{\tau }}^{2}\right)}^{\frac{1}{2}}+\frac{1}{\sqrt{2}}{\left({\tilde{W}}^{T}\tilde{W}\right)}^{\frac{1}{2}})+\lambda_{1}+\lambda_{2}\\ & \leq -\min(\sqrt{2}k_{2},\frac{\sqrt{2}}{2}\delta_{1},\frac{\sqrt{2}}{2}\delta_{2}){\left(\frac{1}{2}\sigma^{2}+\frac{1}{2}{\tilde{\tau }}^{2}+\frac{1}{2}{\tilde{W}}^{T}\tilde{W}\right)}^{\frac{1}{2}}+\lambda_{1}+\lambda_{2}\\ & =-\min(\sqrt{2}k_{2},\frac{\sqrt{2}}{2}\delta_{1},\frac{\sqrt{2}}{2}\delta_{2})V_{1}{}^{\frac{1}{2}}+\lambda_{1}+\lambda_{2} \end{array}$$

According to Lemma 2, Equation (24) is finite-time-stable within $t_{1}\leq \frac{2V_{1}{}^{\frac{1}{2}}(0)}{\theta_{1}\min(\sqrt{2}k_{2},\frac{\sqrt{2}}{2}\delta_{1},\frac{\sqrt{2}}{2}\delta_{2})}$, $0<\theta_{1}<1$, all terms in (17) are bounded. Therefore, ${\tilde{d}}_{m}$ can converge in a bounded set within a finite time; then, the proof is completed. □

In the rest of this section, the finite-time SMC will be introduced.

The sliding surface is defined as follows:

(25) $$S=e(t)+\beta \int_{0}^{t}e(\varphi )d\varphi$$

β represents the integral gain.

According to Lemma 3, ${\hat{d}}_{m}$ will converge to the neighborhood of $d_{m}$ in a finite time, and $\tilde{W}$ and ${\tilde{d}}_{m}$ are bounded. The observed value ${\hat{d}}_{m}$ is then incorporated into the design of the control law to improve the disturbance rejection. The control input is designed as follows:

(26) $$\begin{array}{r} u=\frac{1}{k_{m}}(b_{m}x+{\hat{W}}^{T}h-{\hat{d}}_{m}-a_{1}\mathrm{sgn}S\\ -a_{2}\mathrm{sgn}S+{\dot{x}}_{d}-l_{d}\mathrm{sgn}S-\beta e) \end{array}$$

where $a_{1}$ and $a_{2}$ are positive terms.

**Theorem** **1.** *For the PEA control system (5), Equations (11) and (12) are the adaptive law of RBF-NN weight and disturbance estimation, respectively. Equation (25) is chosen as the sliding surface, and Equation (26) is the designed control input. If *$l_{d}>\left|{\tilde{d}}_{m}\right|$ *and* $a_{1}>\left|{\tilde{W}}^{T}h\right|$*, the control system is bounded.*

**Proof.**  Choose the following Lyapunov function:

(27) $$V_{2}=\frac{1}{2}S^{2}$$

The time derivative of (27) is

(28) $$\begin{array}{ll} {\dot{V}}_{2} & =S\dot{S}\\ & =S(\dot{e}+\beta e)\\ & =S(k_{m}u-b_{m}x-f_{m}+d_{m}-{\dot{x}}_{d}+\beta e)\\ & =S(b_{m}x+{\hat{W}}^{T}h-{\hat{d}}_{m}-a_{1}\mathrm{sgn}S-a_{2}\mathrm{sgn}S+{\dot{x}}_{d}-l_{d}\mathrm{sgn}S-\beta e-b_{m}x-f_{m}+d_{m}-{\dot{x}}_{d}+\beta e)\\ & =S({\hat{W}}^{T}h-{\hat{d}}_{m}-a_{1}\mathrm{sgn}S-a_{2}\mathrm{sgn}S-l_{d}\mathrm{sgn}S-f_{m}+d_{m})\\ & =S({\hat{W}}^{T}h-{\hat{d}}_{m}-a_{1}\mathrm{sgn}S-a_{2}\mathrm{sgn}S-l_{d}\mathrm{sgn}S-W^{T}h+d_{m})\\ & =S(-{\tilde{W}}^{T}h+{\tilde{d}}_{m}-a_{1}\mathrm{sgn}S-a_{2}\mathrm{sgn}S-l_{d}\mathrm{sgn}S)\\ & =-S{\tilde{W}}^{T}h+S{\tilde{d}}_{m}-a_{1}\left|S\right|-a_{2}\left|S\right|-l_{d}\left|S\right|\\ & \leq -a_{2}\left|S\right|\\ & \leq -\sqrt{2}a_{2}V_{2}{}^{\frac{1}{2}}<0 \end{array}$$

Equation (28) is finite-time-stable within $t_{2}\leq \frac{\sqrt{2}V_{2}{}^{\frac{1}{2}}}{\theta_{2}a_{2}}$, $0<\theta_{2}<1$. The convergence time of the PEA system-based SMC is finite, so the proof is completed. □

The saturation function is adopted to replace the sign function (sgn) in (26) and is defined as

(29) $$sat(z)=\{\begin{array}{c} \mathrm{sgn}(z),\\ z/\phi , \end{array}\begin{array}{c} \mathrm{if}\left|z\right|>\phi \\ \mathrm{if}\left|z\right|\leq \phi \end{array}$$

where z is input, and ϕ denotes the boundary layer thickness. The saturation function ensures the sliding surface is always bounded by ϕ.

## 4. Experimental Setup and Results

### 4.1. Experimental Setup

**Figure 1:**
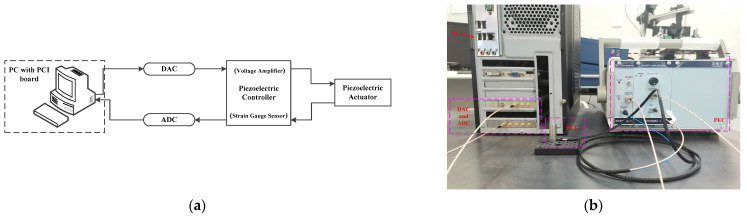
Experimental platform: (**a**) platform setup; (**b**) platform photo.

The diagram of the experimental platform is shown in Figure 1. The platform consisted of a PC with a PCI board, a piezoelectric controller with a voltage amplifier and strain gauge sensor, a PEA with strain gauge sensor, ADC, and DAC. The gain of the piezoelectric controller (PEC) was 15, PEC (E01.B1, Coremorrow, Inc., Harbin, China), which could amplify the input voltage of DAC (PCI-9302, OLP, Inc., Chengdu, China) from 0–10 V to 0–150 V, and the maximum displacement of PEA (PSt20VS12, Coremorrow, Inc.) was 20 µm at input voltage 150 V. The PEA displacement 0–20 µm was transferred by the PEC position sensor as voltage and sampled by ADC (PCI-9203, OLP, Inc.) from 0 to 10 V.

**Figure 2:**
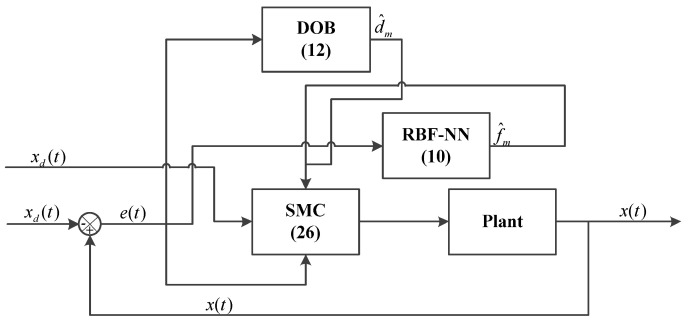
The architecture of the proposed method scheme.

The scheme of the proposed method is shown in Figure 2. The parameters of RBF were selected as $X^{*}=\left[\begin{array}{cc} e & \dot{e} \end{array}\right]$, $\mu ={\left[\begin{array}{cc} 0 & 0 \end{array}\right]}^{T}$, ε=2.0. For the adaptive laws, $\delta_{1}=0.2$, $\delta_{2}=0.25$, $k_{1}=0.5$, $k_{2}=0.3$. The parameters of the controller are designed as β=0.01, $a_{2}=0.1$, $a_{1}=1.0$, $l_{d}=1.2$.

The maximum absolute error and average absolute error are defined as Equations (30) and (31), respectively, and abbreviated as MAE and AAE in this paper.

(30) $$MAE=\underset{1\leq t\leq N}{\max}\left|x_{d}(t)-x(t)\right|$$

(31) $$AAE=\frac{1}{N}\sum_{t=1}^{N}\left|x_{d}(t)-x(t)\right|$$

where $x_{d}(t)$ is the desired displacement, x(t) is the actual displacement generated by PEA, and N is the number of data.

### 4.2. Experimental Verification

To verify the performance of the proposed method, we adopted different control methods, namely [24] (Con.1) and [38] (Con.2) for comparison. The proposed control method and the other methods were all compiled in C language in the VC++ environment. The stiffness and capacitance of the actuator were 60 N/µm and 1.8 µF, respectively. The sampling frequency of the ADC was 200 kHz, and the control voltages calculated using the control method were generated in VC++ and then transferred to the voltage amplifier of PEC through the PCI-9302 card. Experiments were implemented for the following two cases to evaluate the effectiveness of the proposed method: tracking sinusoidal and triangular trajectories. The frequencies of the trajectories were 1, 50, 100, and 200 Hz.

**Figure 3:**
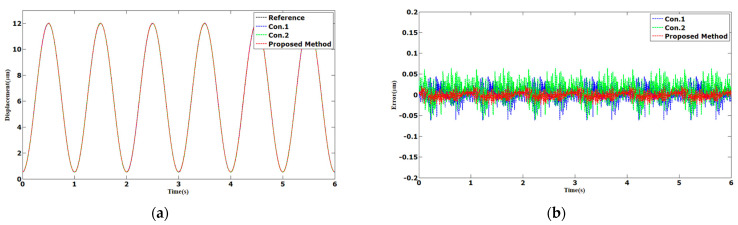
Comparison results of different methods under 1 Hz sinusoidal trajectories: (**a**) displacements; (**b**) errors.

**Figure 4:**
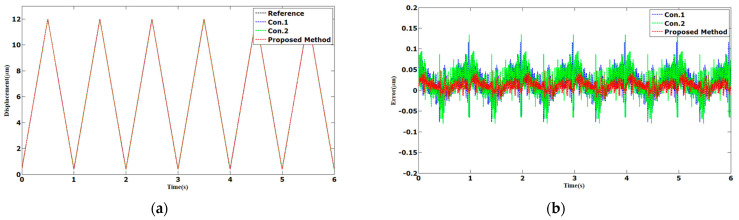
Comparison results of different methods under 1 Hz triangular trajectories: (**a**) displacements; (**b**) errors.

The tracking results of the sinusoidal and triangular trajectories with the 12 µm peak-to-peak amplitude and 1 Hz frequency are shown in Figure 3 and Figure 4. The AAE of the proposed method under sinusoidal and triangular trajectories were 0.0063 and 0.0132 µm, respectively. The MAEs of the proposed method were 0.0238 and 0.0478 µm, which was less than those of Con.1 and Con.2.

**Figure 5:**
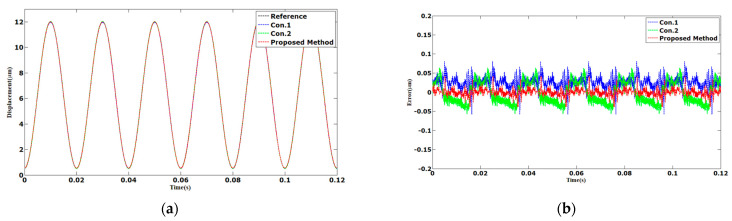
Comparison results of different methods under 50 Hz sinusoidal trajectories: (**a**) displacements; (**b**) errors.

**Figure 6:**
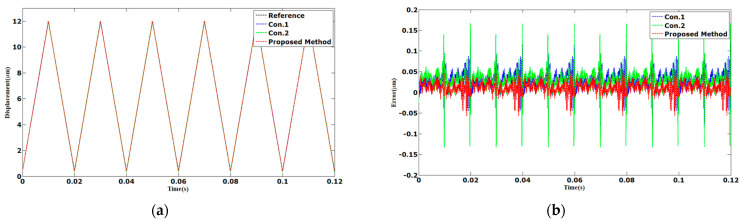
Comparison results of different methods under 50 Hz triangular trajectories: (**a**) displacements; (**b**) errors.

The results of tracking the 50 Hz sinusoidal and triangular trajectories are shown in Figure 5 and Figure 6. The AAE of the proposed method tracking in sinusoidal trajectory was 0.0067 µm. Compared with Con.1 and Con.2, the AAE of the proposed method was reduced by 74% and 73%, respectively. The AAE tracking control in triangular trajectory was reduced by 54% and 59%, respectively, compared with Con.1 and Con.2. The MAEs of the proposed method were 0.0421 and 0.0604 µm, which were less than the other control methods.

**Figure 7:**
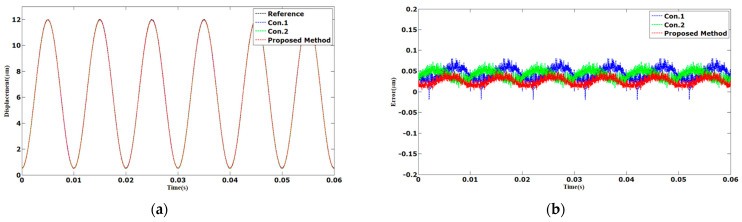
Comparison results of different methods under 100 Hz sinusoidal trajectories: (**a**) displacements; (**b**) errors.

**Figure 8:**
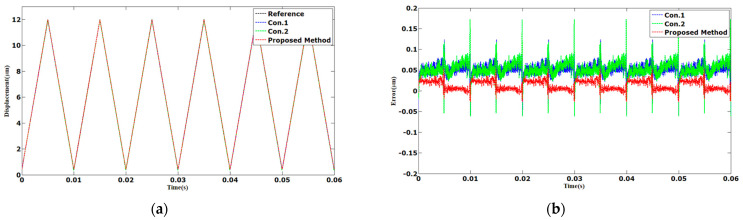
Comparison results of different methods under 100 Hz triangular trajectories: (**a**) displacements; (**b**) errors.

The results of the tracking of 100 Hz sinusoidal and triangular trajectories are shown in Figure 7 and Figure 8. The AAE of the proposed method tracking in sinusoidal trajectory is 0.0257 µm. Compared with Con.1 and Con.2, the AAE of the proposed method was reduced by 41% and 37%, respectively. The AAE tracking control in triangular trajectory was 0.0142 µm. The AAE was reduced by 73% and 74%, respectively, compared with Con.1 and Con.2. The MAEs of the proposed method were 0.0504 and 0.0535 µm. We can observe that the tracking errors of the proposed method were less than those of Con.1 and Con.2.

**Figure 9:**
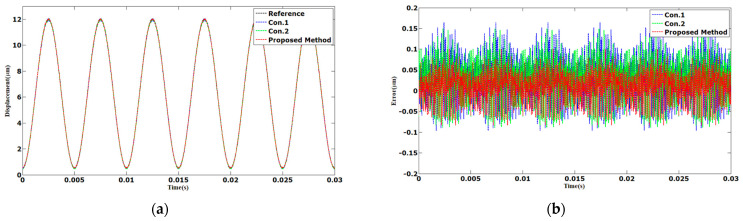
Comparison results of different methods under 200 Hz sinusoidal trajectories: (**a**) displacements; (**b**) errors.

**Figure 10:**
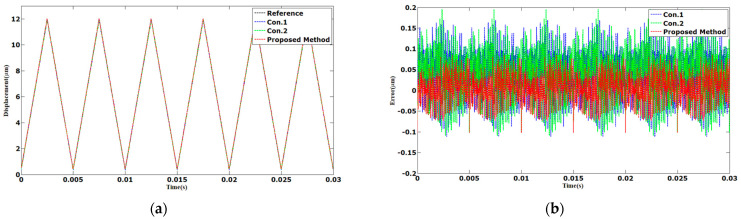
Comparison results of different methods under 200 Hz triangular trajectories: (**a**) displacements; (**b**) errors.

The frequency was further increased to 200 Hz, the sinusoidal and triangular trajectories tracking results are shown in Figure 9 and Figure 10. The AAE of the proposed method tracking in sinusoidal trajectory was 0.0268 µm. Compared with Con.1 and Con.2, the AAE of the proposed method was reduced by 39% and 38%, respectively. The AAE tracking control in triangular trajectory was 0.0261 µm. The AAE was reduced by 50% and 51%, respectively, compared with Con.1 and Con.2. The MAEs of the proposed method were 0.1006 and 0.1025 µm, which were less than Con.1 and Con.2.

For a clear presentation, the AAE and MAE values of the tracking sinusoidal trajectories are listed in Table 1 and Table 2, respectively. Table 3 and Table 4 provide a list of the AAE and MAE values of the tracking triangular trajectories. The control law with finite-time stability in this study can enable the developed control method to achieve better performance than that of the ultimately bounded approach. DOB assists the control method in compensating nonlinear disturbances, especially at high frequencies. The results of the comparative experiments validate that the proposed control method has superior performance in improving the accuracy of tracking the sinusoidal and triangular trajectories under different frequencies. According to the experimental results of tracking control of sinusoidal and triangular trajectories with 200 Hz frequency, the proposed control method can feasibly and effectively realize the high-precision tracking control of the PEA platform under high-frequency conditions.

## 5. Conclusions

In this paper, a finite-time SMC based on the DOB and RBF-NN was proposed. The proposed DOB has the advantage of a simple structure and does not rely on any priori knowledge of disturbances. By using the RBF-NN to replace the hysteresis model of the PEA dynamic system, it is no longer necessary to establish the hysteresis model. The proposed finite-time SMC-based DOB and RBF-NN can ensure that the system errors converge to a small neighborhood of zero in a finite time. The control method with finite-time stability can achieve better performance than that of the ultimately bounded approach. DOB in the control method can compensate for nonlinear disturbances at high frequencies. The experiments of tracking the sinusoidal and triangular trajectories are validated on a commercial PEA platform. The experimental tracking results of the sinusoidal trajectories under the frequencies of 1, 50, 100, and 200 Hz show that the AAE values of the proposed control method were 0.0063, 0.0067, 0.0257, and 0.0268 µm, respectively. In the comparative experiments of tracking control triangular trajectories under the frequencies of 1, 50, 100, and 200 Hz, the AAE values of the proposed hybrid controller were 0.0132, 0.0141, 0.0142, and 0.0261 µm, respectively. All experimental results show that the proposed control method can feasibly and effectively realize the high-precision tracking control of the PEA platform under high and low frequencies.

## Figures and Tables

**Table 1:** AAEs of different methods under desired sinusoidal trajectories with different frequencies.

	1 Hz	50 Hz	100 Hz	200 Hz
Con.1 (µm)	0.0129	0.0261	0.0434	0.0440
Con.2 (µm)	0.0165	0.0255	0.0406	0.0434
Proposed method (µm)	0.0063	0.0067	0.0257	0.0268

**Table 2:** MAEs of different methods under desired sinusoidal trajectories with different frequencies.

	1 Hz	50 Hz	100 Hz	200 Hz
Con.1 (µm)	0.0617	0.0804	0.0804	0.1645
Con.2 (µm)	0.0634	0.0637	0.0724	0.1494
Proposed method (µm)	0.0238	0.0421	0.0504	0.1006

**Table 3:** AAEs of different methods under desired triangular trajectories with different frequencies.

	1 Hz	50 Hz	100 Hz	200 Hz
Con.1 (µm)	0.0258	0.0307	0.0520	0.0526
Con.2 (µm)	0.0287	0.0342	0.0536	0.0553
Proposed method (µm)	0.0132	0.0141	0.0142	0.0261

**Table 4:** MAEs of different methods under desired triangular trajectories with different frequencies.

	1 Hz	50 Hz	100 Hz	200 Hz
Con.1 (µm)	0.1209	0.1166	0.1232	0.1691
Con.2 (µm)	0.1343	0.1664	0.1725	0.1954
Proposed method (µm)	0.0478	0.0604	0.0535	0.1025

## Data Availability

Not applicable.
